# Low pressure (8 mmHg) pneumoperitoneum does not significantly reduce postoperative visual analog scale score after laparoscopic cholecystectomy: Randomized trial

**DOI:** 10.1371/journal.pone.0360476

**Published:** 2026-10-09

**Authors:** Amine Ben Safta, Salsabil Nasri, Imen Samaali, Wajih Dougaz, Raja Bousassi, Hajer Taher, Mehdi Khalfallah, Chadli Dziri, Ramzi Nouira

**Affiliations:** 1 Department of Digestive Surgery B, Charles Nicolle University Hospital, Tunis, Tunisia; 2 Faculty of Medicine of Tunis, University of Tunis El Manar, Tunis, Tunisia; 3 Department of Anesthesiology, Charles Nicolle University Hospital, Tunis, Tunisia; Faculty of Medicine of Monastir, TUNISIA

## Abstract

**Background:**

Low-pressure pneumoperitoneum (LPP) has been proposed to improve postoperative pain in laparoscopic surgery. This randomized controlled trial (RCT) aimed to evaluate the impact of LPP versus standard-pressure pneumoperitoneum (SPP) on postoperative pain and surgical outcomes during laparoscopic cholecystectomy.

**Methods:**

A randomized, controlled, double-blind trial was performed between November 12, 2024 and December 31, 2025. Patients scheduled for elective laparoscopic cholecystectomy (LC) were randomized into two groups: LPP group (8 mmHg) versus SPP group (14 mmHg). The primary outcome was postoperative pain assessed by the Visual Analogue Scale (VAS) at 6 hours. Secondary outcomes included operative time, analgesic consumption, postoperative nausea and vomiting (PONV), and surgical complications. Data were analyzed using an intention-to-treat approach. This trial has been registered at ClinicalTrials.gov (**NCT06685250**) on November 11, 2024.

**Results:**

One hundred sixty consecutive patients were enrolled. There was no statistically significant difference in median VAS pain scores at 6 hours (3 vs. 4; p = 0.221), 12 hours (2 vs. 3; p = 0.074), or 24 hours (1 vs. 2; p = 0.128) postoperatively. However, the mean consumption of paracetamol during the first 24 hours was significantly lower in the LPP group compared to the SPP group (1.04 ± 0.906 vs. 1.35 ± 0.873; p = 0.014). Operative times were similar (59.9 ± 27.5 vs. 58.2 ± 22.4 minutes; p = 0.880). No conversion to laparotomy or major morbidities were recorded.

**Conclusion:**

LPP (8 mmHg) was feasible without increasing operative duration. It did not show a statistically significant reduction in VAS pain scores at 6 hours. Larger studies are required to confirm safety equivalence regarding rare surgical complications.

## Introduction

Laparoscopic cholecystectomy (LC) is widely established as the gold standard treatment for symptomatic gallstone disease [1]. To ensure adequate intra-abdominal visualization and a safe working space, the creation of a carbon dioxide (CO2) pneumoperitoneum is mandatory. In standard clinical practice, insufflation pressures are typically maintained between 12 and 14 mmHg [2].

Despite its benefits, maintaining a standard or high-pressure pneumoperitoneum is not innocuous. Elevated intra-abdominal pressure can cause peritoneal stretching, diaphragmatic irritation, and systemic absorption of CO2, which frequently translate into significant postoperative pain, particularly in the abdomen and shoulder tip [3]. To mitigate these adverse physiological effects, the concept of “low-impact laparoscopy” has gained momentum [4].

While previous meta-analyses have evaluated the use of lower pressures (ranging from 7 to 10 mmHg), the current literature remains controversial due to a high risk of bias, heterogeneous protocols, and varying definitions of low pressure [5–7]. Consequently, there is still no universal consensus on the optimal pressure that balances patient comfort with surgical safety.

We previously published our study protocol detailing methodology to address this gap [8]. This randomized controlled trial (RCT) was designed to test the hypothesis that low-pressure pneumoperitoneum (LPP) at 8 mmHg significantly reduces postoperative pain at the 6th postoperative hour compared with standard-pressure pneumoperitoneum (SPP) at 14 mmHg.

## Methods

### Study design and ethical approval

This was a single-center, prospective, randomized, double-blind, parallel-group clinical trial conducted at department B of general surgery of Charles Nicolle university hospital of Tunis, Tunisia between November 12, 2024 and December 31, 2025.

The trial protocol was published previously in Plos One Journal [8] and registered at ClinicalTrials.gov (NCT06685250) (attached S1 File). Ethical approval was obtained from the institutional review board (approval number FWA 00032748 -IORG0011243) (attached S2 File), and all participating patients provided written informed consent prior to enrollment (attached S3 File).

### Participant selection

Consecutive adult patients scheduled for elective LC were screened for eligibility. **Inclusion criteria** were: age ≥ 18 years with ASA score I or II, and uncomplicated symptomatic gallbladder lithiasis. **Exclusion criteria** were: Choledocholithiasis, converted procedures, history of major upper abdominal surgery, presence of ascites or carcinomatosis, and neurological disorders impairing pain perception.

### Randomization and blinding

Randomization was performed by an independent investigator (CD in the authors’ list) using random permutation tables (9 elements). The randomization was balanced every four patients [9].

To ensure double-blinding, the allocation sequence was concealed in sequentially numbered, opaque, sealed envelopes opened only inside the operating room after the induction of anesthesia. Both the patients and the postoperative independent outcome assessors were strictly blinded to the allocation group. Due to the nature of the intervention and for safety reasons (to allow immediate pressure adjustments if visualization was compromised or hypercapnia occurred), the operating surgeons could not be blinded.

### Surgical Procedure

All procedures were performed by or under the direct supervision of a senior surgeon. Following pneumoperitoneum creation, the intra-abdominal pressure was adjusted according to the allocated group: 8 mmHg for the low-pressure group and 14 mmHg for the standard-pressure group. Surgeons were authorized to adjust the pneumoperitoneum pressure in the event of compromised surgical visualization or cardiopulmonary instability; any such modifications were documented as protocol deviations. At the end of the procedure, residual CO2 was evacuated.

### Outcomes and measurements

- **Primary Outcome:** Postoperative abdominal pain assessed using the Visual Analog Scale (VAS) (0 = no pain, 10 = worst imaginable pain) at 6 hours after surgery
- **Secondary Outcomes:** VAS pain scores at 12 and 24 hours; Postoperative Nausea and Vomiting (PONV) score (0–3 scale); postoperative consumption of pain killer; total operative time; conversion rate; length of hospital stay; and 30-day postoperative morbidity.

### Sample size calculation

Using the VAS scale, the mean value of abdominal pain after LC with a standard pressure of 14 mmHg is estimated in the literature at 4.1 ± 1.8 at 6 hours postoperatively [10]. We hypothesized that patients in the low-pressure group will experience significantly lower postoperative pain compared to those in the standard-pressure group (14 mm Hg). We targeted a minimum clinically important difference in means (Δ) of 1.0 point on the VAS. The sample size was determined according to the method described by Schwartz, Flamant, and Lellouch [9], using the published sample-size reference tables provided on pages 274–275 of their textbook, with a two-sided alpha level of 5% and a statistical power of 90% [9]. Seventy patients per group are needed, i.e., 140 in all. Sample size was overestimated by 20% as per loss of follow-up and/or data discrepancies expectation. An overall sample size of 170 subjects will be enrolled in the study, divided into 85 experimental group vs. 85 placebo group.

### Statistical analysis

Statistical analysis was performed on an intention-to-treat (ITT) basis, ensuring that all randomized patients were analyzed within their originally assigned groups.

Continuous variables were presented as means and standard deviations (SD) or medians with interquartile ranges (IQR) depending on normality. Categorical data were analyzed using the Chi-squared or Fisher’s exact test. Continuous outcomes were compared using the Student’s t-test or Mann-Whitney U test. Analgesic consumption was treated as a discrete count variable (number of paracetamol doses during the first 24 hours). We recorded only the total paracetamol intake per patient. Consequently, we did not record intake at 6, 12, and 24 hours. The comparison between the two groups (“8mm” versus “14mm”) was performed using Student’s t-test.

A p-value < 0.05 was considered statistically significant.

Postoperative VAS scores measured at 6, 12, and 24 hours were additionally analyzed using a linear mixed-effects model to account for within-patient correlation of repeated measurements. Treatment group (8 vs. 14 mmHg), time (6, 12, and 24 hours), and the treatment-by-time interaction were included as fixed effects. Time was modeled as a repeated factor within patients using an unstructured covariance matrix. Restricted maximum likelihood estimation was used. The prespecified between-group comparison of VAS at 6 hours remained the primary efficacy analysis.

Furthermore, no adjustment for multiple comparisons was applied to secondary outcomes; therefore, these findings should be interpreted with appropriate caution given the potential for an increased risk of type I error.

The statistical analysis was conducted using SPSS version 26. Analyses were performed by an independent investigator (CD in the authors’ list) blinded to treatment allocation, ensuring unbiased interpretation of the data.

The completely anonymized minimal dataset underlying these analyses is available in the Supporting Information (attached S4 File).

## Results

### Patient flow and enrollment

During the 14-month study period (November 2024 to December 2025), a total of 317 patients underwent LC for cholelithiasis in the Department B of General Surgery at Charles Nicolle University Hospital of Tunis. Among these, 152 patients were deemed ineligible due to the following exclusion criteria: age under 18 years, an American Society of Anesthesiologists (ASA) score of III, presence of acute cholecystitis, performance of an intraoperative cholangiography, or an associated surgical procedure combined with the cholecystectomy.

Consequently, 165 patients met the eligibility criteria, of whom five declined to participate. A total of 160 patients underwent randomization and were included in the final ITT analysis (n = 80 in the low-pressure group and n = 80 in the standard-pressure group). Two intervention failures occurred in the standard-pressure group, due to hypercapnia, requiring a secondary reduction of the pneumoperitoneum pressure. Fig 1 illustrates the flow diagram of our patients.

**Fig 1:**
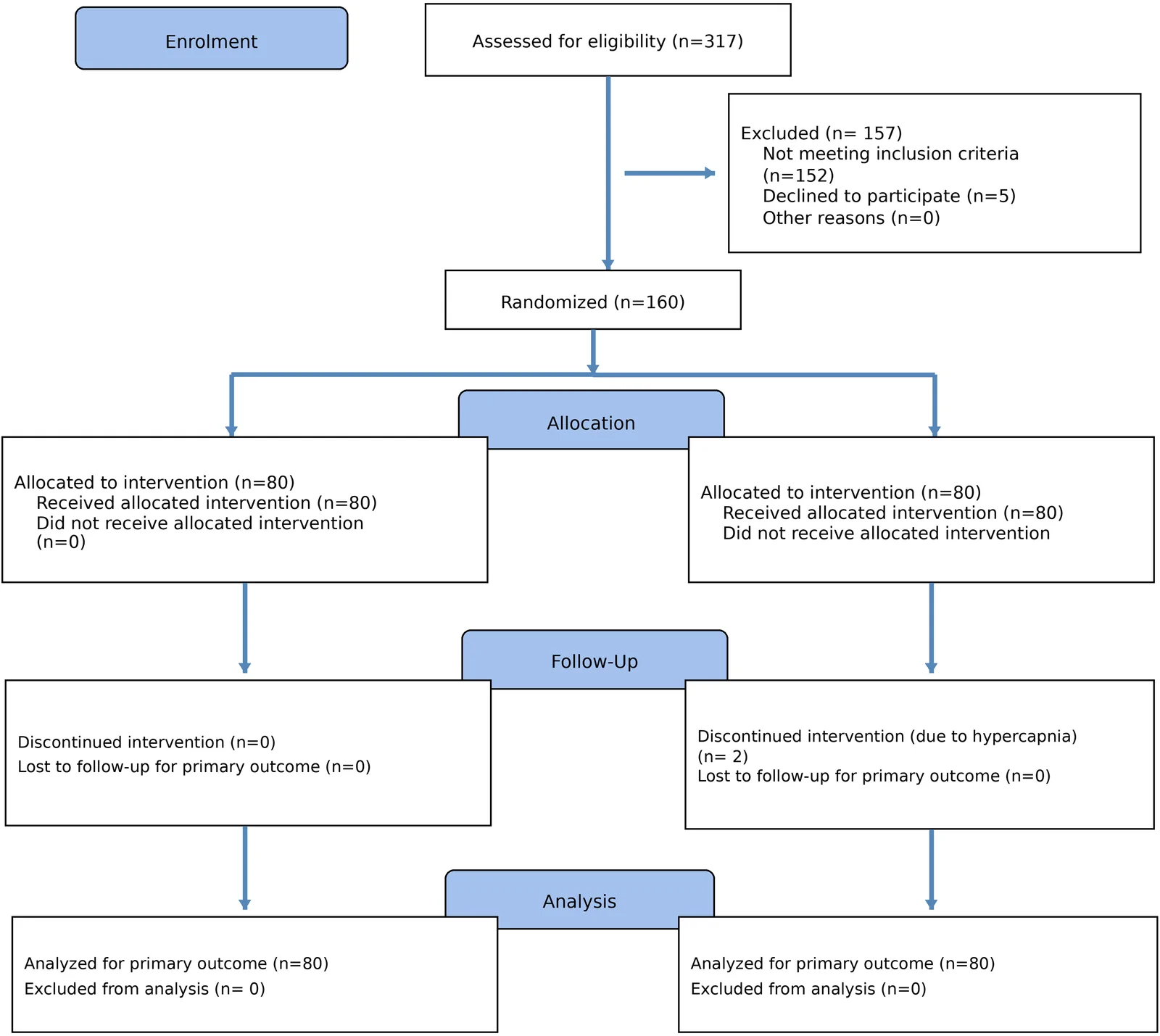
CONSORT flow Diagram.

### Baseline Characteristics

The average age of our patients was 51.5 years (± 15.3). The population was predominantly female, with a sex ratio (M/F) of 0.27. Of 160 patients, 95 patients (59.4%) were classified as ASA I, and 65 patients (40.6%) were classified as ASA II. The mean body mass index (BMI) of our patients was 30.1 ± 4.5 kg/m^2^.

The procedures were performed by a senior surgeon in 71 cases (44.4%) and by a resident under supervision by senior surgeon in 89 cases (55.6%). Cholecystectomy was performed using the standard four-trocar technique in almost all cases (159 patients, 99.4%); a single patient (0.6%) underwent surgery using three trocars. Adhesions were found in 43 patients (26.9%). Intraoperative gallbladder perforation occurred in 44 patients (27.5%). Peritoneal lavage was performed in 57 cases (35.6%). A Redon drain was inserted in 12 patients (7.5%).

Baseline demographic and clinical characteristics were balanced between the two groups, with no statistically significant differences observed, as shown in Table 1.

**Table 1:** Baseline demographic and clinical characteristics of the study population.

Characteristic	Low pressure Group (8 mmHg) (n = 80)	Standard-Pressure Group (14 mmHg) (n = 80)	p-value
Age, years (mean ± SD)	50.6 ± 13.6	51.41 ± 16.7	0.468
Gender, n(%)			0.44
Male	15 (18.8)	19 (23.8)	
Female	65 (81.3)	61 (76.3)	
BMI, Kg/m² (mean ± SD)	30.4 ± 4.6	29.7 ± 4.4	0.245
Respiratory disorders, n(%)			1
No	78 (97.5)	77 (96.3)	
Yes	2 (2.5)	3 (3.8)	
ASA score, n (%)			0.260
I	51 (63.8)	51 (63.8)	
II	29 (36.2)	29 (36.2)	
Preoperative nausea and vomiting, n(%)			0.405
No	76 (95)	78 (97.5)	
Yes	4 (5)	2 (2.5)	
encased calculus on preoperative ultrasound, n(%)			0.681
No	76 (95)	78 (97.5)	
Yes	4 (5)	2 (2.5)	
Operator			0.426
Senior	38 (47.5)	33 (29.4)	
Surgical trainee	42 (52.5)	47 (58.8)	
Peroperative adhesions, n(%)			0.373
No	61 (76.3)	56 (70)	
Yes	19 (23.7)	24 (30)	
Perforation of the gallbladder, n(%)			0.479
No	60 (75)	56 (70)	
Yes	20 (25)	24 (30)	
Peritoneal lavage, n(%)			0.069
No	57 (71.3)	46 (57.5)	
Yes	23 (28.7)	34 (42.5)	
Insertion of Redon drain, n(%)			0.548
No	75 (93.7)	73 (91.3)	
Yes	5 (6.3)	7 (8.7)	

n: effect, SD: standard deviation, BMI: body mass index, ASA: American society of anesthesiologists score

### Primary Outcome: Postoperative Pain at 6 Hours postoperatively

The intensity of postoperative pain, assessed using the VAS, had a median score of 4, with scores ranging from 0 to 9 at 6 hours post-operatively. There was no statistically significant difference between the two groups (LPP versus SPP) (Table 2). Furthermore, a site-specific analysis of pain revealed no statistically significant differences between the cohorts, particularly concerning referred shoulder pain.

**Table 2:** Primary and secondary outcomes.

Characteristic	Low pressure Group (8 mmHg) (n = 80)	Standard-Pressure Group (14 mmHg) (n = 80)	p-value
**VAS pain at 6 hours**, (median, range)	3 [0–9]	4 [0–8]	**0,221**
VAS pain at 12 hours, (median, range)	2 [0–8]	3 [0–8]	**0.074**
VAS pain at 24 hours, (median, range)	1 [0–8]	2 [0–8]	**0.128**
Operative time, minutes (mean ± SD)	59.96 ± 27.51	58.24 ± 22.45	0.880
Number of paracetamol doses during the first 24 hours			**0.014**
median, range	1[0-3]	1[0-3]	
mean ± SD	1.04 ± 0.906	1.35 ± 0.873	
PONV, n(%)	25 (31.3)	33 (41.3)	0.188
Conversion pneumoperitoneum rate, n (%) No	80 (100)	78 (97.5)	0.497
Yes	0 (0)	2 (2.5)	
Length of hospital stay, day (mean ± SD)	1 (0)	1(0)	–
30 day postoperative morbidity rate, n (%)	0	0	–

VAS: visual analogic scale, n: effect, SD: standard deviation, PONV: Postoperative nausea and vomiting.

### Secondary Outcomes

The average duration of the procedure was 59.1 ± 25 minutes. Conversion due to pneumoperitoneum pressure was observed in two patients (1.2%). No conversions to laparotomy were recorded.

The intensity of postoperative pain had a median of 2 with a range of 0–8 at 12 hours post-operatively, and a median of 1 with a range of 0–8 at 24 hours post-operatively. There was no statistically significant difference between the two groups (LPP versus SPP).

In the longitudinal mixed-effects analysis, VAS scores significantly decreased over time (F = 101.189, p < 0.001). However, there was no significant overall effect of pneumoperitoneum pressure (F = 2.550, p = 0.112). The treatment-by-time interaction was also not significant (F = 0.272, p = 0.762), indicating no evidence of a differential postoperative pain trajectory between the 8-mmHg and 14-mmHg groups over the first 24 hours (Fig 2).

**Fig 2:**
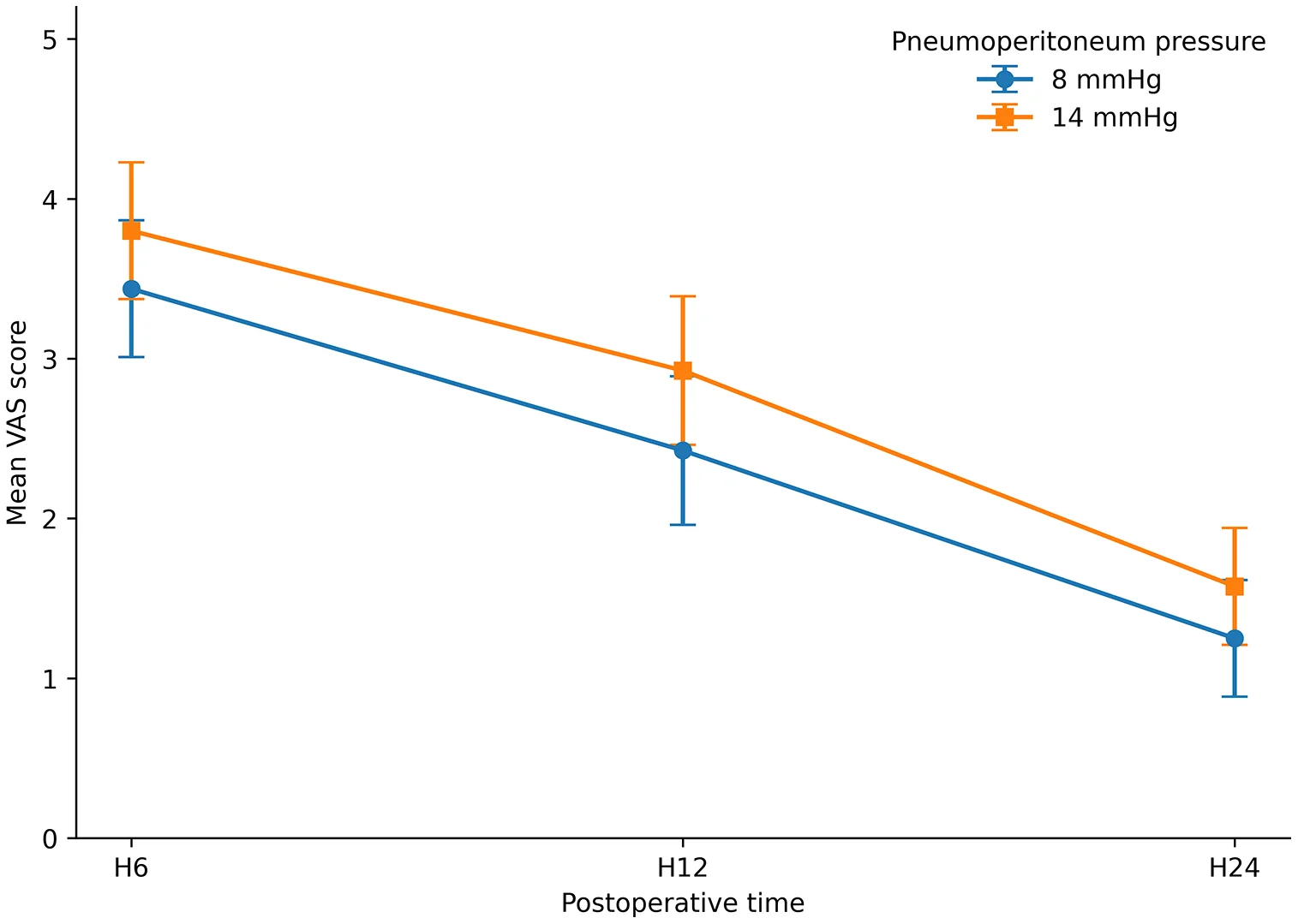
Postoperative pain trajectories according to pneumoperitoneum pressure. Mean VAS scores at 6, 12, and 24 hours after surgery according to pneumoperitoneum pressure group. Error bars represent 95% confidence intervals. The linear mixed-effects model showed a significant effect of time (p < 0.001), with no significant treatment-by-time interaction (p = 0.762).

The number of paracetamol doses during the first 24 hours was lower in the Low-Pressure Group compared to the Standard-Pressure Group (mean ± SD: 1.04 ± 0.91 vs. 1.35 ± 0.87; median [IQR]: 1 [0–2] vs. 1 [1 –2]; p = 0.014) (Table 2).

No analgesics were administered to 34 patients (21.25%). Paracetamol was the most frequently used analgesic, administered to 123 patients (76.9%). Tramadol was used in 22 patients (13.8%), and nefopam (Acupan®) was administered to 3 patients (1.9%).

A combination of two analgesics was prescribed for 23 patients (14.4%): the paracetamol–tramadol combination for 21 patients (13.1%), and paracetamol–nefopam for two patients (1.25%). The mean paracetamol consumption times per 24 hours was 1.04 ± 0.906 for the low-pressure group versus 1.35 ± 0.873 for the control group, with a statistically significant difference (p = 0.014).

Postoperative nausea or vomiting (PONV) was observed in 58 patients (36.3%).

The postoperative course was uneventful for all patients. Table 2 summarized the comparative results of our outcomes.

## Discussion

Absolute pain scores at 6 (main outcome), 12, and 24 hours did not significantly differ between the two groups. The longitudinal mixed-effects analysis further demonstrated a significant decrease in postoperative pain over time in both groups. However, neither the overall treatment effect nor the treatment-by-time interaction was statistically significant, indicating no evidence that low-pressure pneumoperitoneum altered the trajectory of postoperative pain during the first 24 hours compared with standard-pressure pneumoperitoneum. Despite the absence of a significant difference in VAS scores, paracetamol consumption was significantly lower in the low-pressure group. Notably, the use of low-pressure pneumoperitoneum did not increase operative time, with no statistically significant difference between the two groups.

The lack of a statistically significant difference in VAS scores initially appears to contradict existing literature advocating for LPP [7,11]. Although our primary outcome did not demonstrate a statistically significant reduction in postoperative pain, paracetamol consumption during the first 24 hours was lower in the LPP group. Analgesic consumption may provide complementary information to self-reported pain scales, which can be influenced by individual pain perception and psychological factors [12,13]. However, as paracetamol consumption was a secondary outcome and no adjustment for multiplicity was prespecified, this finding should be interpreted cautiously. VAS scores must be interpreted alongside the analgesic consumption data [12]. By operating at 8 mmHg, we minimized the mechanical stretch of the peritoneum and diaphragmatic muscle fibers, thereby reducing the basal nociceptive stimulus [3]. This translates clinically into a reduced reliance on pharmacological pain management.

A persistent apprehension regarding LPP is the potential loss of working space, which could impair the visualization of the hepatocystic triangle and prolong surgery. Our study refuted this concern. The mean operative time showed no statistically significant difference (p = 0.880) between the LPP and SPP groups. These findings are consistent with the RCT conducted by Rashdan et al., despite variations in the operators’ levels of experience [11]. Furthermore, the procedure was successfully completed without conversion to open surgery in all cases. Interestingly, the two instances requiring a pressure adjustment occurred in the standard-pressure group because of hypercapnia, necessitating a secondary reduction in pneumoperitoneum pressure. However, the limited sample size and low-risk population preclude drawing conclusions regarding differences in safety between the two pressure strategies.

In the context of Enhanced Recovery After Surgery (ERAS) pathways, minimizing pharmacological interventions is a relevant objective [14]. Although our protocol primarily relied on paracetamol, the lower paracetamol consumption observed in the LPP group may represent a potential advantage of reducing intra-abdominal pressure. However, given the secondary nature of this outcome and the absence of adjustment for multiplicity, this finding requires confirmation in appropriately powered studies. Implementing LPP as a standard protocol requires no additional equipment and incurs no extra cost, making it an accessible optimization for ambulatory surgeries.

Several limitations must be acknowledged for our study. First, this trial was conducted at a single tertiary institution, which may limit the external validity of our findings regarding varying levels of surgical expertise.

Second, our cohort excluded patients with severe acute cholecystitis. Consequently, these findings are most applicable to elective, uncomplicated symptomatic cholelithiasis.

Additionally, while no major morbidities or conversions to laparotomy occurred in our cohort, the sample size of 160 patients (80 per group) in a low-risk elective population is insufficient to establish safety equivalence or rule out rare intraoperative and postoperative complications between low- and standard-pressure techniques.

Furthermore, while patients and postoperative outcome assessors were strictly blinded, the operating surgeons were inherently aware of the assigned pressure. Although this could theoretically introduce a statistical bias, surgical protocols were highly standardized to mitigate this effect.

Finally, a critical methodological limitation relates to our endpoint design and statistical analysis. We evaluated postoperative pain using the VAS score, a subjective measure, as our primary outcome for which the study was specifically powered. Although we observed a reduction in paracetamol consumption; a highly objective metric; this was only evaluated as a secondary outcome. Because the trial was not powered to assess analgesic use as a primary endpoint, and since no multiplicity adjustments were applied across the various secondary outcomes evaluated, this finding carries a risk of Type I error. Consequently, the reduction in paracetamol consumption should be considered exploratory and hypothesis-generating rather than definitive. Future randomized controlled trials powered specifically on objective criteria, such as analgesic consumption, are needed to corroborate these findings.

## Conclusion

To conclude, in this trial of low-risk elective laparoscopic cholecystectomy, LPP (8 mmHg) was feasible without increasing operative duration. It did not show a statistically significant reduction in VAS pain scores at 6 hours. Larger studies are required to confirm safety equivalence regarding rare surgical complications.

## Supporting information

S1 Checklist Consort_2025_checklist R1. (DOCX)

S1 File Trial Protocol registred at clinicalTrial.gov. (DOCX)

S2 File Study protocol submitted to our Institutional Review Board. (DOCX)

S3 File Informed Consent Form. (DOCX)

S4 File Minimal Dataset (Anonymized underlying data). (XLSX)

S5 File Minimal Dataset (Anonymized underlying data). (XLSX)
