# Supplementary material for: Low pressure (8 mmHg) pneumoperitoneum does not significantly reduce postoperative visual analog scale score after laparoscopic cholecystectomy: Randomized trial
Source: PLoS One. 2026 Oct 9;21(10):e0360476. doi: 10.1371/journal.pone.0360476 (PMC13652247; doi:10.1371/journal.pone.0360476)
Supplement: Supplementary file 3 [file pone.0360476.s003.docx]

**Study protocol submitted to Ethics Commitee of Charles Nicolle University Hospital of Tunis**

**STUDY : LOW-PRESSURE PNEUMOPERITONEUM IN LAPAROSCOPIC CHOLECYSTECTOMY**

**Study protocol**

| **Study title** | Analgesic effect of low-pressure (8 mmHg) CO2 pneumoperitoneum during laparoscopic cholecystectomy. |
| --- | --- |
| **Study name** |  |
| **NCT code** | The protocol will be registered on the ClinicalTrials.gov platform after approval by the Ethics Committee of Charles Nicolle University hospital. |
| **Background** |  |
| **Principal investigator** | **Dr Amine BEN SAFTA**, Assistant Professor of General Surgery, Department of Surgery B, Charles Nicolle Hospital, Tunis |
| **Co-investigators** | **Dr Salsabil NASRI**, Assistant Professor of General Surgery, Department of Surgery B, Charles Nicolle Hospital, Tunis  **Dr Imen SAMAALI**, Associate Professor of General Surgery, Department of Surgery B, Charles Nicolle Hospital, Tunis  **Dr Hajer TAHAR,** Resident in General Surgery, Department of Surgery B, Charles Nicolle Hospital, Tunis  **Dr Med Wajih DOUGAZ**, Associate Professor of General Surgery, Department of Surgery B, Charles Nicolle Hospital, Tunis |
| **Type of study** | Phase III |
| **Study design** | Randomized comparative trial with two parallel groups : “low-pressure (8 mmHg) CO2 pneumoperitoneum during laparoscopic cholecystectomy” versus “standard-pressure (14 mmHg) CO2 pneumoperitoneum”. |
| **Aim** | **Primary aim**: To assess the efficacy of a low-pressure CO2 pneumoperitoneum during laparoscopic cholecystectomy in reducing postoperative pain.  **Secondary aims:**   - To compare the incidence of postoperative vomiting between the two study groups - To compare the incidence of postoperative morbidity between the two study groups |
| **Study population** | Patients with isolated, uncomplicated symptomatic gallstone disease (cholelithiasis) undergoing laparoscopic cholecystectomy in the Department of Surgery B, Charles Nicolle Hospital, Tunis. |
| **Selection criteria** | **Inclusion criteria:**  Patient:   - Male or female, aged over 18 years - ASA class I or II - Consenting patients   Disease:   - Symptomatic gallstone disease - Elective laparoscopic cholecystectomy   **Non-inclusion criteria (preoperative):**  Patient:   - Patients under 18 years of age - Non-consenting patients - ASA class III or IV   Disease:   - Complicated gallstone disease (acute cholangitis, cholecystitis, pancreatitis) - Associated common bile duct stones - Pathology associated with gallstone disease   **Failure criteria:**   - Patients in whom a transcystic drain was left in place - Conversion to open surgery - Associated intra-abdominal procedures |
| **Study treatments** | According to the randomization arm, during the procedure (laparoscopic cholecystectomy) patients will receive either:   - Insufflation of a low-pressure (8 mmHg) CO2 pneumoperitoneum after trocar insertion at standard pressure - Insufflation of a standard-pressure (14 mmHg) CO2 pneumoperitoneum throughout the procedure |
| **Data collected** | Selection criteria  Sociodemographic characteristics  Preoperative clinical parameters  Preoperative laboratory parameters  Intraoperative findings  Study treatment (allocation)  Postoperative clinical follow-up and investigations |
| **Outcome measures** | **Primary endpoint:** Abdominal and interscapular (shoulder-tip) pain at 6 hours postoperatively, assessed using the Visual Analog Scale (VAS).  **Secondary endpoints**:   - Abdominal and interscapular pain at 12 and 24 hours postoperatively - Postoperative vomiting within 24 hours - Postoperative morbidity up to 30 days postoperatively - Postoperative length of hospital stays |
| **Follow-up schedule** | Several follow-up visits will be carried out:   - Inclusion visit to verify patient eligibility and enroll the patient if the selection criteria are met - In-hospital period, during which several pain assessment visits will be performed at H6, H12 and H24 - Outpatient follow-up visit between postoperative days 10 and 15 |
| **Study duration** | Enrollment period: 12 months  Follow-up period: 1 month |
| **Randomization** | Patients will be randomized in a 1:1 ratio to either the “low-pressure” group (8 mmHg) or the “standard-pressure” group (14 mmHg).  Randomization will be performed by an independent investigator (Dr DZIRI) using random permutation tables (blocks of 9). The balance of randomization will be checked every four patients. |
| **Sample size** | Based on the VAS, the mean postoperative abdominal pain score after laparoscopic cholecystectomy is estimated in the literature at 4.1 ± 1.8 (H6). With a power of 90%, a significance level of 5% and a two-sided comparison between the two study arms, 70 patients per group are required, i.e. 140 in all. Sample size will be overestimated by 20% as per loss of follow-up. An overall sample size of 170 subjects will be enrolled in the study, divided into 85 experimental group vs. 85 placebo group. |
| **Assessment of the primary endpoint** | A systematic assessment of postoperative pain at H6 will be performed using the VAS. This assessment will be conducted in a double-blind manner: the assessor evaluating postoperative pain will be unaware of the patient's group allocation. |
| **Protocol violation** | A protocol violation is defined for any enrolled patient in whom:   - A randomization allocation error occurred - Pain assessment was performed outside the 6th postoperative hour |
| **Time windows and intervals** | The study dates and durations are defined as follows:   - The inclusion date is the day the patient is selected and meets all inclusion and non-inclusion criteria - The operation date is the day the surgical procedure is performed - The study start date is the date the randomization envelope is opened - The clinical assessment (VAS) of the primary endpoint corresponds to H6 after the end of the procedure |
| **Statistical analysis plan** | - Definition of the analysis populations and description of baseline parameters - Assessment of the comparability of the two groups - Analysis of the primary endpoint - Analysis of the secondary endpoints |
| **Ethics** | Approval from the Ethics Committee of Charles Nicolle Hospital will be sought before the start of the study. |
| **Communication and publication** | Results to be published in a surgical journal. |
