# Supplementary material for: Low pressure (8 mmHg) pneumoperitoneum does not significantly reduce postoperative visual analog scale score after laparoscopic cholecystectomy: Randomized trial
Source: PLoS One. 2026 Oct 9;21(10):e0360476. doi: 10.1371/journal.pone.0360476 (PMC13652247; doi:10.1371/journal.pone.0360476)
Supplement: Supplementary file 4 [file pone.0360476.s004.docx]

**PATIENT INFORMATION AND CONSENT FORM**

I. **Study Title:**

Analgesic effect of low-pressure CO₂ pneumoperitoneum (8 mmHg) during laparoscopic cholecystectomy.

II. **Objective of the Study:**

To evaluate the effect of low-pressure CO₂ pneumoperitoneum (8 mmHg) during laparoscopic cholecystectomy on the reduction of abdominal pain.

III. **Patient Information**

**Name:** .......................................................
**Surname:** .......................................................
**Date of Birth:** .......................................................
**Phone:** .......................................................
**Address:** .......................................................
**Patient study number:** .......................................................

After reviewing the objectives of the study, I declare the following:

I have been clearly and fully informed about the aims, conditions, and stages of the study.
I was granted sufficient time to reflect and decide freely about my participation.
I was given the opportunity to ask all necessary questions to ensure full understanding.

I have been informed that my decision to participate or not in this study is entirely voluntary, and that I may withdraw from the study at any time, simply by notifying the investigator, without any prejudice to the quality of care or medical services I receive.

I acknowledge that this study has obtained the approval of the Medical Ethics Committee on the date of ................................, in accordance with applicable laws and regulations.

I understand that this research does not impose any particular treatment or medication change on me or my attending physician unless I choose to do so.

I have been informed that my personal information may be used for research purposes, in a strictly confidential manner, and will only be accessed by authorized individuals under the supervision of the principal investigator.

I agree that the principal investigator, or their delegate, may handle the data collected in this study in an anonymous and confidential manner.

I have been informed that, upon completion of this study, I will be informed of the overall results by my attending physician once they become available.

I understand that my consent does not release the investigators or the official sponsor of the study from any of their legal or ethical responsibilities, and that I retain all my rights under the law.

IV. **For the Investigator**

**Investigator’s name:** .......................................................
**Phone number:** .......................................................
**Date:** .......................................................
**Signature:** .......................................................

V. **For the Patient**

I freely agree to participate in this study under the terms described above, after reading and approving them.

**Date:** .......................................................
**Signature:** .......................................................

VI. **For the Witness**

I, the undersigned ....................................................... (Name and Surname), hereby declare that I have no relation to the investigator or the study sponsor.
I certify that ....................................................... (Name and Surname of the patient) has clearly and explicitly expressed their consent to participate in the study.

**Date:** .......................................................
**Signature:** .......................................................

**If the patient is unable to sign, please specify the reason:**
..................................................................................................................
..................................................................................................................
